# Association between essential hypertension and bone mineral density: a systematic review and meta-analysis

**DOI:** 10.18632/oncotarget.20325

**Published:** 2017-08-18

**Authors:** Ziliang Ye, Haili Lu, Peng Liu

**Affiliations:** ^1^ Guangxi Medical University, Nanning, Guangxi 530021, China; ^2^ Department of Cardiology, The First Affiliated Hospital of Guangxi Medical University, Nanning, Guangxi 530021, China; ^3^ Department of Anatomy, Guangxi Medical University, Nanning, Guangxi 530021, China

**Keywords:** association, essential hypertension, bone mineral density, meta-analysis

## Abstract

**Background:**

We conducted this systematic review and meta-analysis to evaluate the association between essential hypertension (EH) and bone mineral density (BMD).

**Results:**

17 articles were included in our meta-analysis, with a total of 39,491 patients. Of these, 13,375 were patients with EH and 26,116 were patients without EH. Meta-analysis results showed that EH can reduce the BMD of the lumbar spine (95% CI: −0.08∼0.01, P=0.006), femoral neck (95% CI: −0.09∼-0.02, *p* = 0.001), ward's triangle (95% CI: −0.45∼-0.25, p=0.000), femoral intertrochanteric (95% CI: −0.90∼-0.64, *p* = 0.000), calcaneus (95% CI: −0.31∼-0.18, *p* = 0.000) and distal forearm (95% CI: −0.09∼-0.03, *p* = 0.000), but EH cannot reduce the BMD of the femur rotor (95% CI: −0.07∼0.24, *p* = 0.273). Subgroup analysis showed that EH can reduce the BMD of the lumbar spine (95% CI: −0.11∼-0.03, *p* = 0.000) and femoral neck (95% CI: −0.11∼-0.07, *p* = 0.000) in Asian populations. In non-Asian populations, EH can reduce the BMD of the femoral neck (95% CI: 0.04∼0.19, *p* = 0.002), but cannot reduce the BMD of the lumbar spine (95% CI: −0.04∼0.11, *p* = 0.346).

**Materials and Methods:**

We conducted a systematic review of the published literature on the association of EH and BMD by searching the Cochrane Library, PubMed, EMBASE, CBM, CNKI and VIP databases inception to October 2016. Stata 11.0 software was used for data analysis.

**Conclusions:**

Our meta-analysis suggests that EH can reduce the BMD of the human body, and for different parts of the bone, the degree of reduction is different. In addition, for different regions and populations, the reduction level of BMD is inconsistent.

## INTRODUCTION

With society's trending toward aging and unhealthy lifestyle changes (such as high heat, high fat food intake increase and reduction in physical activity), the prevalence rate of essential hypertension (EH) and osteoporosis (OP) increases every year, and these have become two of the most common diseases in the world [1–3]. Research has shown that the number of fractures related to osteoporosis has reached approximately 1500000 each year in the United States [4, 5]. In China, the population of people over 60 years old has been estimated as high as one hundred thirty-two million, and of these, approximately 90000000 patients have been diagnosed with osteoporosis [6, 7]. Osteoporosis fracture is a serious consequence of osteoporosis that can lead to the increased risk of mortality and morbidity. In addition, treating and nursing patients with osteoporosis and osteoporosis fracture requires not only an investment in manpower and material resources but is also expensive. This high cost is not conducive to a society's goal of a harmonious and stable development; thus, identifying risk factors to prevent osteoporosis has become one of the hot issues.

Numerous studies have indicated that age, gender, smoking, drinking coffee, coronary heart disease, diabetes, essential hypertension and decreased estrogen levels are risk factors for osteoporosis [8–10]. Hypertension is one of the common diseases found in the clinic. Research results have shown that hypertension and osteoporosis are a common occurrence. Both hypertension and osteoporosis are age-related diseases and result from the interaction of genetic and environmental factors. However, there is still a controversy concerning whether a correlation exists between hypertension and osteoporosis. Several studies [11, 12] have indicated that hypertension is negatively correlated with bone mineral density. Cappuccio F P [11] and his colleague conducted a prospective study of 3676 women, and they found that high blood pressure in elderly white women is associated with increased bone loss at the femoral neck. This association may reflect greater calcium losses associated with high blood pressure, which may contribute to the risk of hip fractures. Similarly, Yang S and his colleagues published a longitudinal study in 2014 with 1,032 men and 1,701 women aged 50 years and older who had participated in the Dubbo Osteoporosis Epidemiology Study. Their results found that women with hypertension had lower BMD at the femoral neck (0.79 versus 0.82 g/cm(^2^)) than those without hypertension. After adjusting for confounding factors, hypertension was an independent risk factor for fragility fracture (HR: 1.49; 95% CI: 1.13–1.96). In men, hypertension was associated with higher femoral neck BMD (0.94 versus 0.92 g/cm(^2^)), but no significant association was found between hypertension and fracture risk. Alternatively, some studies [13, 14] have suggested that there is no correlation between essential hypertension and bone density. Fahad Javed F and his colleagues conducted a retrospective, cross-sectional study with 965 participants. The result found that the proportion of patients with both osteopenia and osteoporosis was similar in those with and without hypertension (osteopenia: 50% versus 50%, *p* = .95; osteoporosis: 18% versus 19%, *p* = 0.95).

Therefore, we conducted this meta-analysis to evaluate the relationship between essential hypertension and bone mineral density and to provide a theoretical basis for early prevention of osteoporosis.

## RESULTS

### Literature search

**Figure 1:**
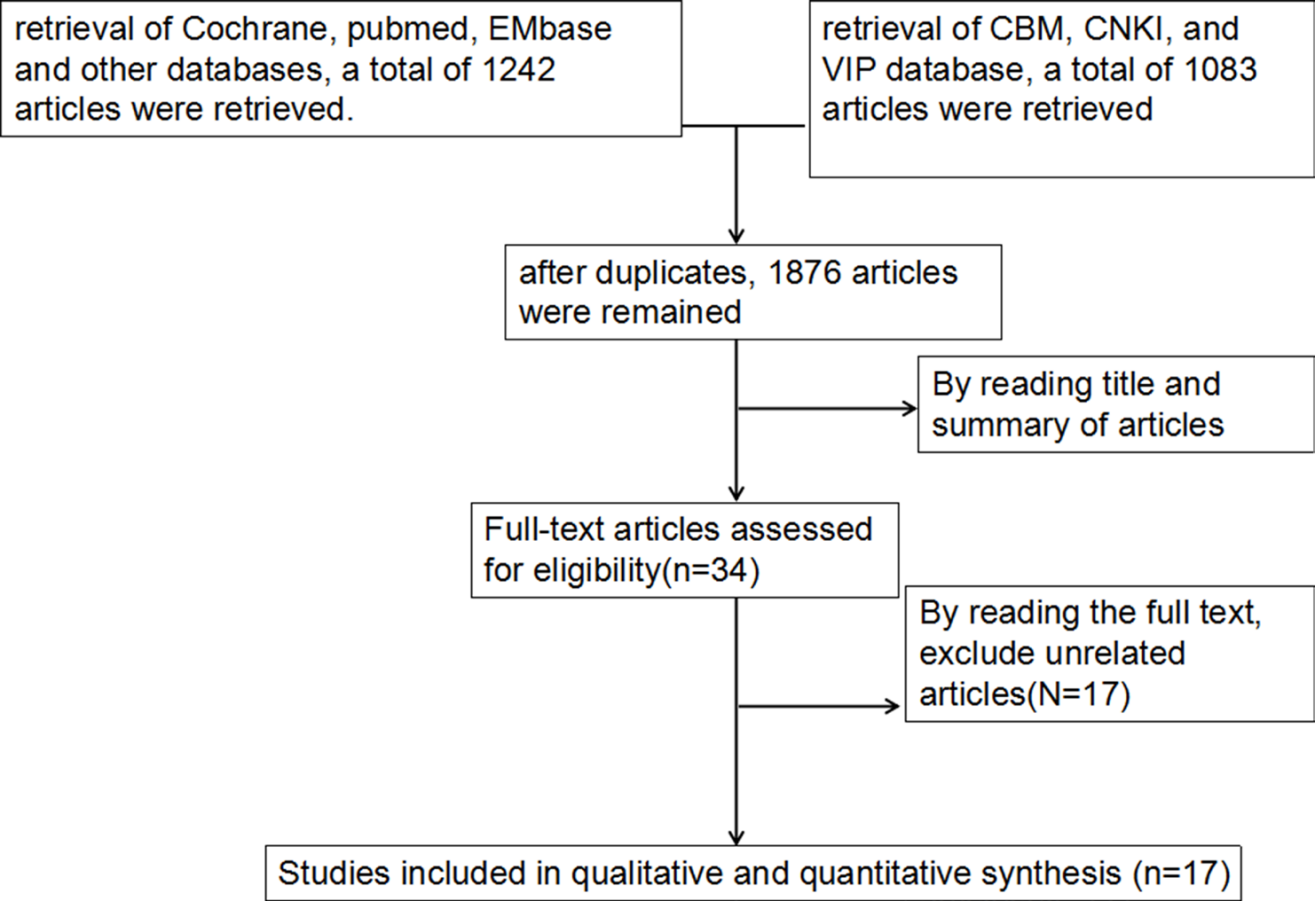
Flow diagram of selection of studies included in the meta-analysis

Following the development of our search strategy, 2325 articles were retrieved. After excluding the duplicates and articles that did not meet the inclusion criteria, we reviewed 34 possible relevant studies in full-text. A total of 17 studies [12–28] were excluded for the following reasons: six were narrative reviews, four were not related to the outcome of interest, one described the same population, and six reported the association. Finally, a total of 17 articles were included in our meta-analysis (Figure 1).

### Study characteristics

Table 1 shows the descriptive data for all 17 included studies, representing a total of 39,491 patients, Of this total, 13,375 were patients with essential hypertension, and 26116 were patients without essential hypertension. These studies were published from 2001 to 2015. In each study, the number of essential hypertension patients ranged from 13 to 6,636 and the number of patients without essential hypertension ranged from 13 to 18,195 without essential hypertension. Thirteen studies were conducted in China, one in the North America, one in Australia and one in Korea.

**Table 1:** Characteristic of case-control studies included in the meta-analysis

researcher	year	country	hypertension (yes/no)	Male/female	age	measuring position	measuring instrument
Chen K^15^	2009	China	138/324	0/462	Population of 40-80 years old	lumbar spine (1-4), femoral neck, large femoral rotor, Ward's zone	Dual energy X-ray
He L^16^	2008	China	103/135	0/238	Population of 30-80 years old	lumbar spine, femoral neck, Ward's zone, Large femoral rotor	Dual energy X-ray
Wang QP^17^	2009	China	34/16	0/50	Population of 74.0±13.8 years old	lumbar spin, femoral neck	Dual energy X-ray
Lin QM^18^	2011	China	37/21	58/0	Population of 60-83 years old	lumbar spine (2-4), femoral neck, Large femoral rotor, Ward's zone	Dual energy X-ray
Liu Y^19^	2013	China	78/104	unclear	Population of Over 60 year old	lumbar spine, femoral neck, Ward's zone, oblique eminence of cuboid bone	Dual energy X-ray
Lu DH^20^	2015	China	82/80	unclear	Population of 61-71 years old	lumbar spine, whole body	Dual energy X-ray
Yan LY^21^	2011	China	13/28	unclear	Population of 52-64 years old	lumbar spin (1-4), femur	Dual energy X-ray
Wang X^22^	2001	China	121/90	108/103	Population of 32-65 years old	lumbar spin (1-4), femoral neck, Ward's zone, oblique eminence of cuboid bone	Dual energy X-ray
xiang H^23^	2011	China	311/291	293/309	Population of 44-79 years old	lumbar spin (L2-L4), femoral neck, Ward's zone, oblique eminence of cuboid bone	Dual energy X-ray
Yue RR^24^	2014	China	256/205	unclear	Population over 40 years of age	lumbar spin (L1-L4)	Dual energy X-ray
Javed^13^	2012	America	631/334	0/965	Women over 65 years of age	lumbar spin (L1-L4), femoral neck	Dual energy X-ray
Yang^11^	2014	Australia	660/2093	1032/1701	People over 50 years of age	lumbar spin, femoral neck	Dual energy X-ray
Tsuda^25^	2001	Japan	31/14	0/45	Women over 55 years of age	lumbar spin (L2-L4)	Dual energy X-ray
Lee^26^	2015	Korea	4089/4350	unclear	Population over 50 years of age	femoral neck	Dual energy X-ray
Perez^14^	2003	Spain	82/40	0/122	postmenopausal women of 36-76 years old	lumbar spin (L2-L4)	Dual energy X-ray
Xu JR^27^	2003	china	60/60	60/60	Population of 63.6±10.2 years old	forearm distal	Dual energy X-ray
Shi DZ^28^	2012	china	115/91	206/0	Population of 64.87±3.4 years old	forearm distal	Dual energy X-ray

### Quality evaluation of included studies

The methodological quality of the included studies was generally good. The NOS scores ranged from five to eight (Table 2). The median NOS score was 6.05.

**Table 2:** Quality assessment of eligible studies based on newcastle-ottawa scale

researcher	Selection				Comparability	Exposure			Scores
	Adequate difinition of cases	Representativeness of the cases	Selsction of controls	Definition of controls	Control for important factor	Ascertainment of exposure	Same method of ascertainment for cases and controls	Non-Responserate	
Chen K^15^	☆	☆	☆	☆	☆	-	☆	-	6
He L^16^	☆	☆	☆	☆	☆	-	☆	☆	7
Wang QP^17^	☆	☆	☆	☆	☆	☆	-	-	6
Lin QM^18^	☆	☆	☆	☆	☆	-	☆	☆	7
Liu Y^19^	☆	☆	-	☆	☆	☆	☆	☆	7
Lu DH^20^	☆	☆	-	☆	☆	-	☆	☆	6
Yan LY^21^	☆	☆	☆	-	☆	☆	-	☆	6
Wang X^22^	☆	☆	-	☆	☆	☆	☆	-	6
xiang H^23^	☆	☆	-	-	☆	☆	-	☆	5
Yue RR^24^	☆	☆	☆	☆	-	☆	☆	☆	7
Javed^13^	☆	☆	☆	☆	☆	☆	☆	☆	8
Yang^11^	☆	☆	☆	☆	☆	☆	☆	-	7
Tsuda^25^	☆	☆	-	☆	☆	☆	☆	☆	7
Lee^26^	☆	☆	☆	☆	☆	☆	☆	☆	
Perez^14^	☆	☆	☆	-	☆	☆	☆	☆	7
Xu JR^27^	☆	☆	☆	-	-	☆	☆	-	5
Shi DZ^28^	☆	☆	☆	☆	☆	☆	-	-	6

### Lumbar spine and femur rotor

**Figure 2:**
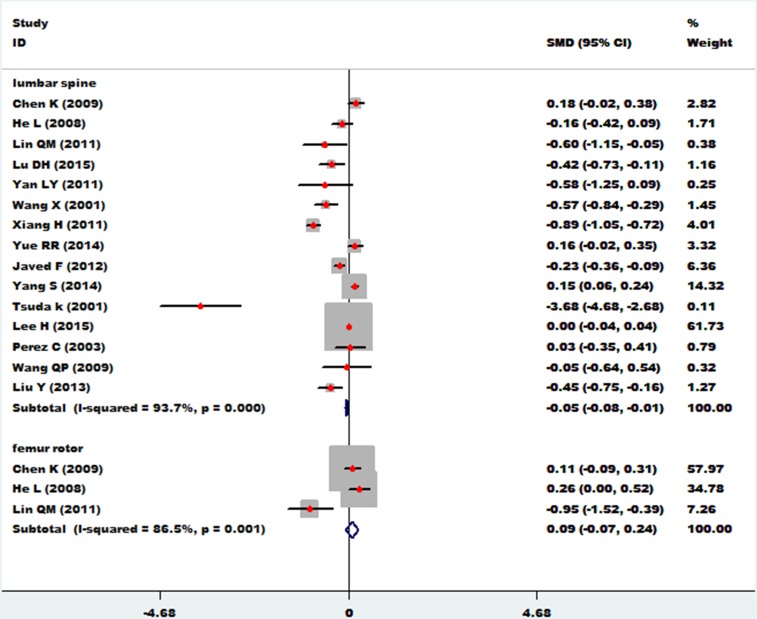
Forest plot of bone mineral density of lumbar spine and femur rotor with essential hypertension

**Figure 3:**
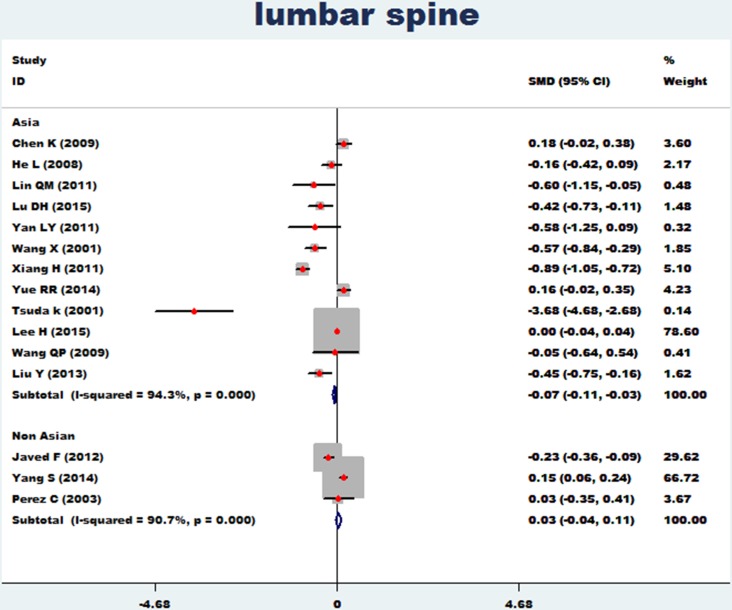
Forest plot of bone mineral density of lumbar spine with essential hypertension (Asian and non-Asian)

The association between essential hypertension and bone mineral density of the lumbar spine was investigated in 15 studies [11–27]. The combined standard mean difference (SMD) was −0.05 (95% CI: −0.08∼0.01, *P* = 0.006), with significant heterogeneity (*P*_for heterogeneity_ = 0.000; I^2^ = 93.7%). The association between essential hypertension and bone mineral density of the femur rotor was investigated in 3 studies [15, 16, 18]. The combined standard mean difference (SMD) was 0.09 (95% CI: −0.07∼0.24, *P* = 0.273) with significant heterogeneity (*P*_for heterogeneity_ = 0.001; I^2^ = 86.5%) (Figure 2). In subgroup and sensitivity analysis of the lumbar spine (Asian and non-Asian), essential hypertension obviously reduced bone density of the lumbar spine (95%CI: −0.11∼−0.03, *p* = 0.000) in Asian populations, but showed no association with bone density of the lumbar spine in non-Asian populations (95% CI: −0.04∼0.11, *p* = 0.346) (Figure 3).

### Femoral neck and ward's triangle

**Figure 4:**
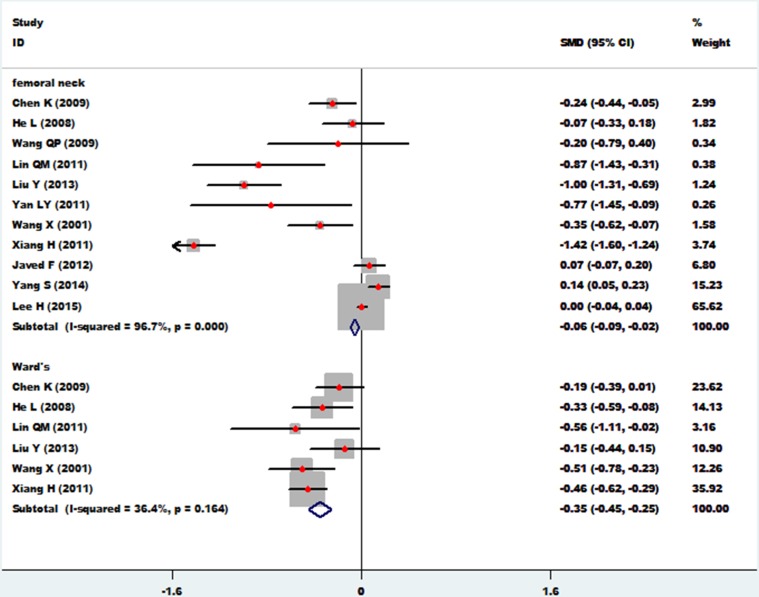
Forest plot of bone mineral density of femoral neck and Ward's triangle with essential hypertension

**Figure 5:**
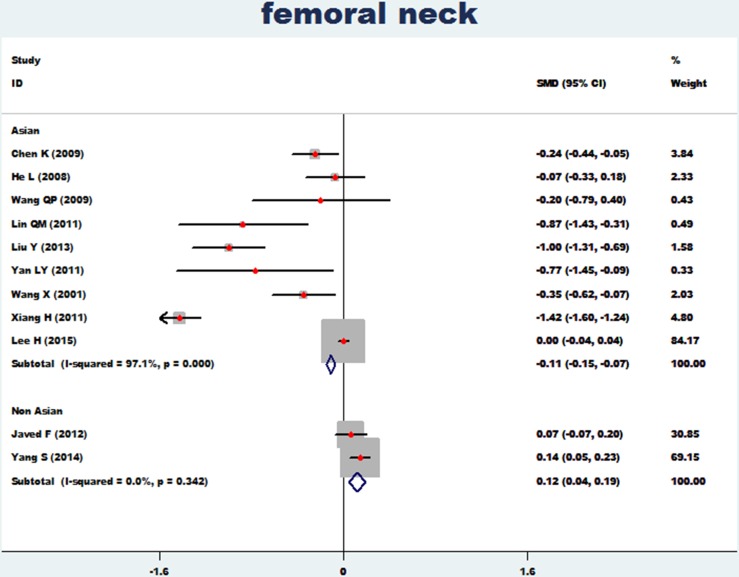
Forest plot of bone mineral density of femoral neck with essential hypertension (Asian and non-Asian)

The association between essential hypertension and the bone mineral density of femoral neck was investigated in 11 studies [12, 13–19, 21–23, 26]. The combined standard mean difference (SMD) was −0.06 (95%CI: −0.09∼−0.02, *p* = 0.001), with significant heterogeneity (*P*_for heterogeneity_ = 0.000; I^2^ = 96.7%). The association between essential hypertension and bone mineral density at Ward's triangle was investigated in 6 studies [15, 16, 18, 19, 22, 23]. The combined standard mean difference (SMD) was −0.35 (95% CI: −0.45∼−0.25, *p* = 0.000), with small heterogeneity (*P*_for heterogeneity_ = 0.164; I^2^ = 36.4%) (Figure 4). In subgroup and sensitivity analysis of the femoral neck (Asian and non-Asian), essential hypertension obviously reduced bone density of the femoral neck in Asian populations (95% CI: −0.15∼−0.07, *p* = 0.000) and non-Asian populations (95% CI: −0.15∼−0.07, *p* = 0.002) (Figure 5).

### Femoral intertrochanteric, calcaneus and forearm distal

**Figure 6:**
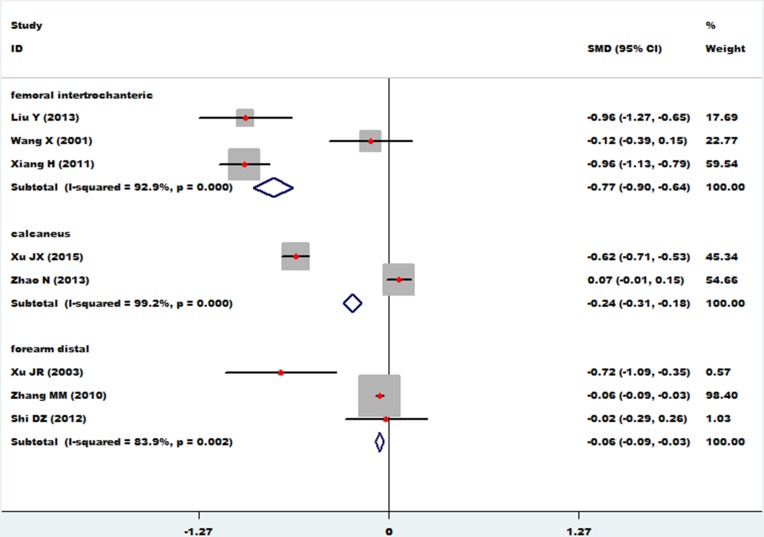
Forest plot of bone mineral density of femoral intertrochanteric, calcaneus, and distal forearm with essential hypertension

The association between essential hypertension and bone mineral density of the femoral intertrochanteric region was investigated in 3 studies [19, 22, 23]. The combined standard mean difference (SMD) was −0.77 (95% CI: −0.90∼−0.64, P= 0.000), with significant heterogeneity (*P*_for heterogeneity_ = 0.000; I^2^ = 92.9%). The association between essential hypertension and bone mineral density of the calcaneus was investigated in 2 studies [27, 28]. The combined standard mean difference (SMD) was −0.24 (95% CI: −0.31∼−0.18, *P* = 0.000), with significant heterogeneity (*P*_for heterogeneity_ = 0.000; I^2^ = 99.2%). The relationship between essential hypertension and bone mineral density of the distal forearm was investigated in 3 studies [14, 27, 28]. The combined standard mean difference (SMD) was −0.06 (95% CI: −0.09∼-0.03, *P* = 0.000), with significant heterogeneity (*P*_for heterogeneity_ = 0.002; I^2^ = 83.9%) (Figure 6). Because of the limited number of studies, we failed to conduct subgroup and sensitivity analyses on these associations.

### Publication bias

**Figure 7:**
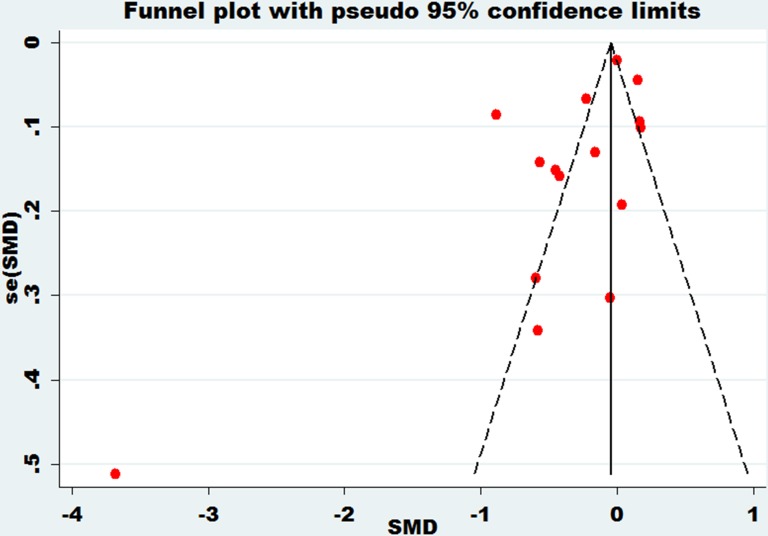
Funnel plot of bone mineral density of lumbar spine with essential hypertension

**Figure 8:**
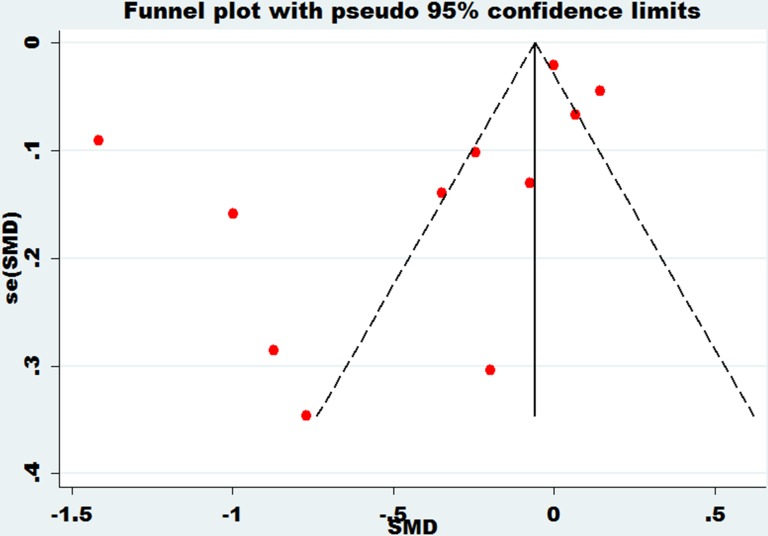
Funnel plot of bone mineral density of femoral neck with essential hypertension

**Figure 9:**
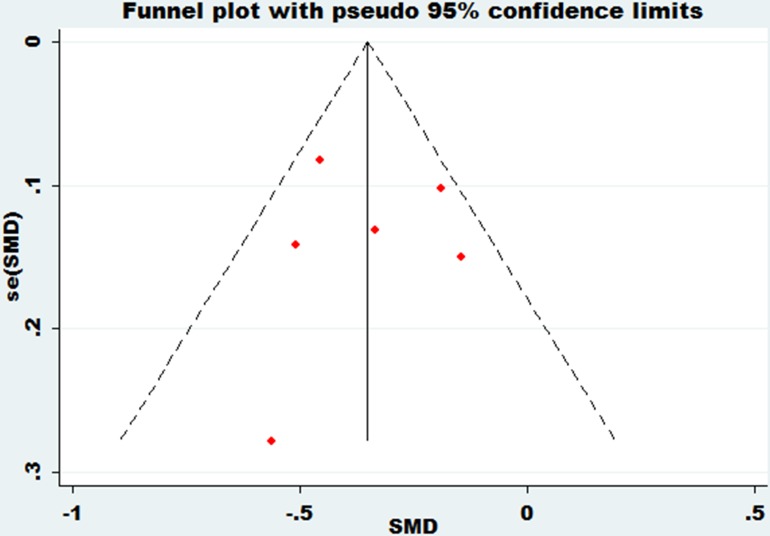
Funnel plot of bone mineral density at Ward's triangle with essential hypertension

Funnel plots were performed on the large heterogeneity of the results. The funnel plot of bone mineral density of the lumbar spine and essential hypertension is shown in Figure 7. The funnel plot of bone mineral density of the femoral neck and essential hypertension is shown in Figure 8. The funnel plot of bone mineral density at Ward's triangle and essential hypertension is shown in Figure 9. As we can see from the funnel plot above, there is a certain bias in the articles included. Next, a meta-regression was conducted. Egger's test was used to show that no significant publication bias was found (Table 3).

**Table 3:** Publication bias (Egger's test)

	Std. Err	*t*	*P*	95% CI
lumbar spine	1.281335	−2.20	0.047	[−5.584, −0.048]
femoral neck	2.152489	−1.82	0.102	[−8.789, 0.948]
femur rotor	3.229358	−1.68	0.342	[−46.445, 35.619]
Ward’s	1.779999	−0.02	0.982	[−4.983, 4.900]
femoral intertrochanteric	9.907325	0.59	0.663	[−120.079, 131.690]
forearm distal	2.083957	−0.87	0.545	[−28.288, 24.669]

## DISCUSSION

In contemporary society facing acceleration of population aging, the incidence of osteoporosis (OP) has been increasing every year [29, 30]. OP is a systemic, metabolic disease that exhibits primary clinical manifestations of low back pain and decreased activity [31, 32]. Patients with severe osteoporosis may even appear to have a short height, which places them at risk of spinal deformity and fragility fractures. Additionally, the pain caused by osteoporosis can cause serious decline of patient's daily life quality. Skeletal deformation, as well as rising pulmonary infection rates, can cause disability and significantly increase mortality. At present, the clinical treatment of OP primarily, uses the method of drug therapy to intervene. However, there is currently no specific drug to reverse the development of OP [33, 34]. Therefore, early prevention is one of the fundamental methods to treat OP. In search for the early prevention methods for osteoporosis, researchers have conducted a large number of experimental studies [35, 36]. In current research, one of the hot-button topics is how to identify the risk factors in the early stages OP. Epidemiological studies have observed a large number of risk factors for OP, such as old age, low body mass, coronary heart disease, diabetes and poor blood glucose control, high blood ALP, essential hypertension and low estrogen levels.

Our study used meta-analysis to evaluate the relationship between essential hypertension and bone mineral density. Our results showed that essential hypertension can significantly reduce bone mineral density of the human body, including the lumbar spine (95% CI: −0.08∼0.01), femoral neck (95% CI: −0.09∼−0.02), Ward's triangle (95% CI: −0.45∼−0.25), femoral intertrochanteric region (95% CI: −0.90∼−0.64), calcaneus (95% CI: −0.31∼−0.18) and distal forearm (95% CI: −0.09∼-0.03), but not including the femur rotor (95% CI: −0.07∼0.24). Our meta-analysis results are consistent with Shi DZ [28] and Gotoh. Gotoh [37] studied the relationship between bone density, blood pressure and serum osteocalcin. The results showed that the bone density of hypertensive patients was significantly lower than that of the control group, and the relationship between bone density and systolic blood pressure was negative (r=-0.385). Furthermore, the results indicated that essential hypertension might be a factor that causes decreasing bone density.

Our study also found that essential hypertension had different effects on bone mineral density at different sites. The mechanism may be that ① different parts of body differ in skeletal muscle strength and exhibit inconsistent activity, which leads to varied effects on bone mineral density at different sites [38–40]. Ward's triangle is primarily composed of cancellous bone, has a local blood supply that is poor, and is prone to fracture; therefore, Ward's triangle is a sensitive region for detecting bone mineral density. The main function of the lumbar spine is to bear weight of the body. Because of this, the local calcium and phosphorus shape is good; thus, the bone density is less affected by high blood pressure. The femur rotor plays an important role in the process of standing and walking, so the local blood supply is good, which is favorable for promoting active osteoblasts and reducing the activity of osteoclasts. ② Antihypertensive drugs have different effects on different target organs. Many research results show that calcium channels exist not only in vascular smooth muscle, cardiac muscle and skeletal muscle cell membrane but also exist in skeletal muscle cells [41–45]. With antihypertensive drugs, calcium antagonists can also act on calcium channels in skeletal cells when they are acting on calcium channels in the vascular smooth muscle cells. Calcium channels of both skeletal muscle cells and cardiac muscle cells are L-type, which are voltage-dependent channels. All of these channels can be blocked by calcium antagonists and then affect the calcium metabolism of bone cells. Conducting an experiment in rabbits, Durieze [46] showed that nifedipine can cause cancellous bone loss, a decline in epiphyseal bone formation, decreased mineral deposition rate, and bone plate thinning. However, human studies showed that compared with BMD (lumbar [L2-L4], femur, radius), bone metabolic markers (including alkaline phosphatase, urine calcium/creatine, oxoprolinase/creatine) and regulating hormones (including testosterone, PTH, 1,25 (OH) 2D3, calcitonin), there have no significant difference between the nifedipine group and the control group [47, 48]. Therefore, further trials are needed to confirm these results.

In this study, we investigated the corresponding reasons for the heterogeneity and carried out a subgroup analysis and meta-regression, Egger's test showed that no significant publication bias was tested (all *P* > 0.05). Thus, the results of this systematic meta-analysis are highly reliable.

## MATERIALS AND METHODS

### Literature search

A comprehensive literature search was performed to identify articles about the significance of essential hypertension and bone mineral density. The PubMed, EMBASE, Cochrane Library, CNKI, CBM and VIP databases (last update October 2016) were used to search for relevant articles with the following combination of search terms: “Bone Densities”, “Density, Bone”, “Bone Mineral Density”, “Bone Mineral Densities”, “Density, Bone Mineral”, “Bone Mineral Content”, “Bone Mineral Contents”, “Osteoporoses”, “Osteopenia”, “bone loss”, “Hypertension”, “Blood Pressure, High”, “Blood Pressures, High”, “High Blood Pressure” and “High Blood Pressures”. To expand our search, the bibliographies of articles that remained after the selection process were screened for additional suitable studies.

### Study selection

Inclusion criteria for studies were as follows: (i) conducted on essential hypertension and bone mineral density in the last ten years; (ii) divided participants into a case group and a control group; (iii) reported the specific value of bone density or could be obtained by calculating the reported OR and 95% CI; (iv) published or accessible before October 2016; (v) selected participants in accordance with the WHO/ISH essential hypertension diagnostic criteria; (vi) did not exclude participants on the basis of gender; and (vi) measured bone mineral density using dual-energy x-ray absorptiometry.

Two reviewers (Ziliang Ye and Haili Lu) independently reviewed the titles and abstracts of the retrieved articles, applying the inclusion and exclusion criteria mentioned above. Articles were rejected if they were clearly ineligible. These two reviewers then independently reviewed the full-text versions of the remaining articles to determine their eligibility for inclusion. Disagreements were resolved by consensus.

### Data extraction

Two reviewers (Ziliang Ye and Peng Liu) independently extracted the relevant data from each article and recorded these data on a standardized form. Any difference was resolved by consensus. The following data were extracted from these studies: an animal experiment; a cross-sectional study of the literature; a review, letters, and other non-essential literature; a non-randomized case-control study; information is not complete; OR and 95% CI values are not reported; and bone mineral density was measured by ultrasonic bone density instrument.

### Quality assessment

Two reviewers (Ziliang Ye and Haili Lu) independently assessed the risk of bias using the Newcastle-Ottawa Scale (NOS), which consists of three factors: patient selection, comparability of the study groups, and assessment of outcome. A score of 0–9 (represented by stars) was allocated to each study. The studies achieving six or more stars were considered to be of high quality.

### Statistical analysis

The odds ratio (OR) with a 95% confidence interval (CI) was used to express the pooled effect on discontinuous variables. The summary estimates of continuous variables were presented as standard mean differences (SMD) with 95% CI. Heterogeneity was quantified using the I^2^ statistic, where I^2^ > 50% represented between-study inconsistency. When there was no between-study inconsistency, fixed-effects meta-analyses were conducted to pool these outcomes across the included trials. When heterogeneity existed, the random-effects model was used. Publication bias was evaluated using a funnel plot. The results were considered statistically significant if *P* < 0.05. The pooled analyses were performed with Stata 11.0 software.

### Limitations

There are several limitations in this study. (1) A total of 17 studies were included in this study, and most of these studies have some limitations. This study's inclusion criteria resulted in more studies concentrated in Asia, and a small portion of trials from other areas. Under these circumstances, our findings may only apply to Asian populations and is not applicable to other populations due to genes, the environment, or diet. (2) The studies included in this meta-analysis did not clearly report the patients’ stage of hypertension or whether they were taking antihypertensive agents. (3) The sample size of this study was small, and the patient population mainly came from Asian countries, so some associations could not undergo subgroup analysis by country or nation.

In our meta-analysis, we found that essential hypertension may be a risk factor for osteoporosis. However, due to the quantity and quality of the included literature, confirmation of this conclusion still requires further.
